# Patients’ perceptions of waiting for bariatric surgery: a qualitative study

**DOI:** 10.1186/1475-9276-12-86

**Published:** 2013-10-18

**Authors:** Deborah M Gregory, Julia Temple Newhook, Laurie K Twells

**Affiliations:** 1 Faculty of Medicine, Memorial University, 300 Prince Philip Drive, St. John’s, Newfoundland and Labrador, Canada; 2 Eastern Health Regional Authority, St. John’s, Newfoundland and Labrador, Canada; 3 School of Pharmacy, Memorial University, 300 Prince Philip Drive, St. John’s, Newfoundland and Labrador A1B 3V6, Canada

**Keywords:** Bariatric surgery, Equity, Grounded theory, Wait time, Health services research, Access

## Abstract

**Background:**

In Canada waiting lists for bariatric surgery are common, with wait times on average > 5 years. The meaning of waiting for bariatric surgery from the patients’ perspective must be understood if health care providers are to act as facilitators in promoting satisfaction with care and quality care outcomes. The aims of this study were to explore patients’ perceptions of waiting for bariatric surgery, the meaning and experience of waiting, the psychosocial and behavioral impact of waiting for treatment and identify health care provider and health system supportive measures that could potentially improve the waiting experience.

**Methods:**

Twenty-one women and six men engaged in in-depth interviews that were digitally recorded, transcribed verbatim and analysed using a grounded theory approach to data collection and analysis between June 2011 and April 2012. The data were subjected to re-analysis to identify perceived health care provider and health system barriers to accessing bariatric surgery.

**Results:**

Thematic analysis identified inequity as a barrier to accessing bariatric surgery. Three areas of perceived inequity were identified from participants’ accounts: socioeconomic inequity, regional inequity, and inequity related to waitlist prioritization. Although excited about their acceptance as candidates for surgery, the waiting period was described as stressful, anxiety provoking, and frustrating. Anger was expressed towards the health care system for the long waiting times. Participants identified the importance of health care provider and health system supports during the waiting period. Recommendations on how to improve the waiting experience included periodic updates from the surgeon’s office about their position on the wait list; a counselor who specializes in helping people going through this surgery, dietitian support and further information on what to expect after surgery, among others.

**Conclusion:**

Patients’ perceptions of accessing and waiting for bariatric surgery are shaped by perceived and experienced socioeconomic, regional, and waitlist prioritization inequities. A system addressing these inequities must be developed. Waiting for surgery is inherent in publicly funded health care systems; however, ensuring equitable access to treatment should be a health system priority. Supports and resources are required to ensure the waiting experience is as positive as possible.

## Introduction

According to WHO, obesity, defined as a body mass index (BMI) ≥ 30 kg/m^2^, is a serious global health concern and is reaching epidemic proportions. In 2011,13.1% of Canadian adults had Class I obesity (BMI 30.0 -34.9 kg/m^2^), 3.6% had Class II obesity (BMI 35.0 -39.9 kg/m^2^), and 1.6% had Class III obesity (BMI ≥ 40.0 kg/m^2^) [1]. Healthcare professionals are concerned about increases in BMI ≥ 30 kg/m^2^ due to its correlation with chronic conditions (e.g., cardiovascular disease, hypertension, diabetes mellitus, some cancers) [2-5], its adverse psychosocial consequences (e.g., social bias, prejudice, discrimination, psychological effects) [6-8], reduced quality of life [9] and its relationship with premature mortality [10].

The increase in human body size is far from straightforward, and the focus on obesity tends to conflate body size with health and fitness [11-14]. Common measurement methods are highly problematic [15], particularly when applied at the individual level [16,17]. However, in the health care system they are often used to define eligibility for obesity non-surgical and surgical management and treatment [18].

Non-surgical management options include diet and exercise programs and lifestyle modifications. However, these options are rarely effective in attaining long-term weight loss, and have even been demonstrated to cause weight gain [19]. In recent years, bariatric surgery has become increasingly viewed as a preferred “treatment” option. Eighty percent of bariatric surgery patients are women [20,21]. Criteria for this elective surgery are BMI ≥ 35 kg/m^2^ with a co-morbid condition such as type II diabetes, or BMI ≥ 40.0 kg/m^2^[18]. The surgery is associated with significant and sustainable weight loss, substantial improvements in obesity-related diseases, particularly type II diabetes and improvements in quality of life [22,23]. Additionally, studies have reported a reduction in the relative risk of death from 35% to 89% [24-28].

Quantitative and qualitative evidence exists on the impact of waiting for elective surgery from the patients’ perspective with a focus on maximum tolerance, quality of life, and the nature of the waiting experience for common elective general surgical procedures (cataract, joint replacement, among others) [29]. In Canada, the wait times for bariatric surgery are long – the longest of any elective procedure - with patients reportedly dying while waiting for surgery [30]. A small number of studies provide evidence on the impact of waiting for elective bariatric surgery from the patients’ perspective. The available evidence from pre-surgical qualitative studies has focused on the experience of choosing bariatric surgery, the exploration of the expectations and beliefs about obesity and treatment and the impact of bariatric surgery [31], the meaning of awaiting bariatric surgery [32], the change process experienced by patients undergoing bariatric surgery prior to surgery and at years 1 and 2 post-surgery [33], and how an empowerment program for morbidly obese individuals involving a course in lifestyle change, bariatric surgery, and aftercare can work on an individual’s identity [34,35]. The studies are comparable in terms of the reasons provided for choosing bariatric surgery as a treatment option for morbid obesity (i.e., health and social), the issue of lack of control over eating, and the assertion that bariatric surgery is a life-saving option and last resort. In an earlier article from this study [36] we explored participants’ perspectives on their histories of weight gain, focusing on their explanations for weight gain as well as emotions surrounding their weight gain experiences, with particular attention given to gender-based differences and gendered discourses.

The purpose of this study was to describe the patients’ meanings of “waiting for weight loss surgery” in the province of Newfoundland and Labrador (NL) and to develop a greater understanding of participants’ support needs during this period. The primary objective was to develop an understanding of the pre-surgical experience of patients that choose to undergo a surgical weight loss intervention for the management of morbid/clinical obesity after being placed on a waitlist for bariatric surgery. The meaning of bariatric surgery and the psychosocial impact of waiting for this form of treatment for individuals must be understood if multidisciplinary bariatric clinical team providers are to act as facilitators in promoting satisfaction with care and quality care outcomes. In a publicly funded healthcare system that promotes universal care, this research is highly relevant for policy makers who want to ensure that patients have equal access to treatment based on need. In this paper we focus on patients’ experiences while waiting, particularly the emotional consequences of waiting, and the insights that these experiences bring to a discussion of equity, including socioeconomic, regional, and waitlist prioritization inequities. We include participants’ recommendations on how the waiting experience can be made more positive.

## Methods

### Setting

In 2011, Eastern Health, the largest regional health authority in NL, began providing bariatric surgery to the residents of the province.

### Study design

Grounded theory methodology involving the simultaneous collection, categorization and analysis of data was initially used [37,38]. Data for this study were collected between June 2011 and April 2012. This study is one of a number of research initiatives of the Translational Research Program in Bariatric Care at the Faculty of Medicine at Memorial University, St. John’s, Newfoundland and Labrador [39]. Phase 2 of this study, a post-surgical qualitative interview, is in progress and is focused on patients’ perceptions of their physical, emotional, and psychosocial health and well-being and definitions of “success” twelve months post surgery.

### Ethical concerns

Ethical approval for this study was given by the university’s ethics approval board. If necessary, arrangements were made to refer the interviewee to the multidisciplinary bariatric surgery team nurse practitioner or family doctor for counselling.

### Population

The target population was individuals assessed by a surgeon or nurse practitioner in the bariatric surgery clinic of a large tertiary care hospital in Newfoundland and Labrador and deemed eligible for surgery based on clinical practice guidelines. 27 of 51 (53%) eligible patients approached agreed to participate in the study.

### Participants

A purposive sample of twenty-seven bariatric patients, including twenty-one women and six men, participated. This non-probability sample of participants ranged from 26 to 64 years, with an average age of 45.3 years. Participant demographics are presented in Table 1. Six participants (22%) had a high school education or less, 15 (56%) had some post-secondary education, and 6 (22%) had a university degree. The majority of participants were Caucasian (26, or 96%, while 1 identified as aboriginal), married or living with a common-law partner (18, or 66%), had children (21, or 78%), and were working full-time (16, or 59%). Approximately 85% of the sample reported three or more co-morbid conditions. BMI data were not collected during the interview since all participants had been categorized with a BMI ≥ 35 kg/m^2^ with co-morbid conditions, or BMI ≥ 40 kg/m^2^, met the Canadian consensus guidelines for eligibility for criteria for bariatric surgery, were approved for bariatric surgery by the bariatric surgeon, and at the time of the initial interview, were waiting for bariatric surgery. The average BMI of the bariatric surgery cohort that had already received surgery at our centre was 46.3 kg/m^2^ (range 35.7- 58.4) [40]. Participants’ self-identified waiting periods at the time of the interview varied widely, with one third waiting for less than six months, and half waiting for more than five years. The waiting time varied because a bariatric program was slated to start around 2000, although the start-up of the multidisciplinary bariatric surgery clinic did not occur until May 2011 when designated funding was made available.

**Table 1:** Demographic characteristics of the sample (N = 27)

**Demographic characteristic**	**Frequency**	**%**
Sex		
Female	21	77.8
Male	6	22.2
Marital Status		
Married	18	66.7
Divorced/separated	4	14.8
Single	3	11.1
Widowed	2	7.4
Employment status		
Full-time	16	59.3
Part-time	1	3.7
Unemployed	1	3.7
Retired	2	7.4
Disability	3	11.1
Full-time homemaker	4	14.8
Education		
High School (completed or some)	6	22.8
College (completed or some)	15	55.6
University (completed or some)	6	22.2
# Self-reported comorbid conditions		
0	4	14.8
1	8	29.6
2	3	11.1
≥ 3	12	44.4
Age (years) ( mean, range)	45.3 (26 to 64)	

## Data collection

Data for this study were collected between June 2011 and April 2012. After initial contact by the surgeon or nurse practitioner, interested individuals were given a cover letter, brief study summary, and consent form by the research nurse coordinator, and re-contacted to schedule interviews. The interviews were conducted by the second author (JTN). Prior to the start of each interview, the participants were once again informed about the purpose of the study, that participation was voluntary, and that they had the right to withdraw at any time. Informed written consent was obtained from each participant prior to the interviews, including their consent to the use of digital audio recordings. Assurances were made that confidentiality would be maintained at all times and that anonymity would be preserved during publication of the results. Two semi-structured interviews were conducted with each participant: the first initial in-depth interview took place either in-person or by telephone, as per the preference of the participant. Each initial interview lasted 60 to 90 minutes. Interviews included the collection of demographic data including age, sex, marital status, employment status, occupation, self-reported medical history, educational level, time waiting for surgery. An interview guide was used and other questions were added that evolved from the thematic analysis [see interview guide questions]. Questions focused on the length of time waiting for surgery and experiences with health care providers and the health care system, in general. Additional questions evolved from the thematic content analysis (weight loss expectations following surgery). The semi-structured nature of the interview allowed participants to share insights that they were not explicitly asked about (cycle of obesity, choosing bariatric surgery and the decision-making process, social supports). The transcriptions of the interviews were done by a professional transcriptionist. The interviewer compared the paper transcripts to the digitally recorded interviews to insure accuracy. The paper transcripts did not contain any identifiable information.

### Interview guide

I am interested in your experiences while waiting for bariatric surgery. I would like for you to take some time to reflect upon these experiences and tell me in your own words what waiting for bariatric surgery means for you. You can share any thoughts, feelings, and ideas about your experiences. Feel free to talk about whatever comes to mind.

1. How long have you been waiting for bariatric surgery?

2. Tell me about your experiences and your decision making process to have weight loss surgery. How did you decide to have this surgery? Was there something specific that you were feeling or experiencing prior to making the decision (probe – potential health issues). Could you describe what it was like for you then? What supports helped you during your decision-making process?

3. Could you think back to when you were first accepted for surgery and describe what it was like for you then?

4. Thinking back to the time before you were accepted, could you describe what it was like for you then? Could you describe any changes you have experienced since you were accepted?

5. How did others respond to you after you made your decision to have weight loss surgery?

6. How would you rate the care you received while waiting? Could the care be improved? Any other supports you need?

7. How are things going for you today? How have you been feeling lately?

8. What have you been told about the surgery?

9. What are your expectations for after the surgery? How much weight do you expect to lose?

10. Have you talked to others about the surgery? What are their reactions?

Any other comments or thoughts you would like to share?

## Data analysis

The study began with a grounded theory approach, designed to build theory from participants’ accounts. As the research progressed, a “hybrid inductive-deductive approach” was developed. This approach combined the inductive methods of grounded theory, including constant comparative analysis [37,38], with feminist methods that emphasize the importance of reflexivity [41,42]. The four guiding principles of this approach were as follows: (1) inclusion of as wide a variety of participants as possible; (2) participation of participants in developing the focus of the research; (3) valuing participants’ embodied knowledge; and (4) focus on the complexity and diversity of participants’ perspectives and experiences.

Constant comparative analysis guided the generation and treatment of data. Data collection, coding and analysis occurred simultaneously. A software program was not used to code the transcribed interviews; instead, coding was performed on the paper copies. Three levels of coding were used to examine the data: open, axial and selective [37,38]. Working independently, two coders (first and second authors) initially read the transcripts and broke the data down into codes (open coding), re-read transcripts and identified and coded emerging categories and themes (axial coding), while exploring similarities and differences amongst them and between individuals and groups of individuals (e.g., females versus males). Categories and themes were linked into theoretical constructs. The two coders worked together to collapse the categories into a set reflective of participants' experiences of waiting for bariatric surgery and the importance attached to them. Regular sessions were held to discuss major themes and to identify the conceptual categories and properties generated by the initial joint coding and analysis. Possible relationships between and among major categories were identified and presented as a model that represents the factors related to the patients’ perspectives of waiting for bariatric surgery (selective coding). Theoretical sampling was used during data collection and analysis to test the data for representativeness. Participants were deliberately selected according to theoretical needs and direction of the research by the research team which determined the final sample size. Post-interview field notes, memos, graphic illustrations and diagrams, and theoretical notes were also used during the process of analysis [37,38].

A qualitative study is credible when the participants recognize the descriptions and interpretations of the experience as their own [43,44]. Each participant was asked to review and confirm an interpretive summary of his/her transcript during a follow-up interview. This interview was conducted by telephone one to three months later, and lasted about 20 minutes. This step was also necessary to understand the importance of each category and property for grasping the waiting experiences of participants. In this interview, the interviewer read the summary to the participants and gave them an opportunity to comment on and identify any gaps in the interpretation and provide additional information that may have been forgotten. Final confirmation of the interpretive summary was provided by all participants, ensuring validity of the conclusions that were drawn. The emerging categories, properties, and descriptors were validated through an independent examination. An external reviewer who was not part of the team, but experienced in grounded theory methodology and the constant comparative method of analysis, was commissioned to perform this independent review. This process resulted in a more refined set of themes and codes. For the current paper the data were subjected to re-analysis to identify barriers to accessing bariatric surgery.

## Results

This paper focuses on one major construct of the substantive theory which deals with patients’ perceptions of inequity and the impact of waiting for bariatric surgery. Participants’ perceptions highlight the emotional consequences of waiting for surgery as well as draw attention to issues of socioeconomic, regional, and waitlist inequity. Health care provider and health system supportive measures identified by participants that could potentially improve the waiting experience are also discussed.

### Perceived socioeconomic inequity

Participants’ accounts of waiting for bariatric surgery often touched on socioeconomic inequity. Many participants had explored private surgical options, but were discouraged to find out how expensive the surgery would be. The cost was well beyond what most lower- or middle-income participants could afford as expressed in the following quotations.

I was really disappointed. [The doctor] told me that it was $10,000… which I couldn’t afford and it wasn’t covered by MCP [Medical Care Plan, provincial health care insurance]. … He told me that people were going away to [city in another province] and such places to get it done if they really wanted it done. I was just after getting divorced at the time. I came out of it in tears. … I’ve waited all this time and now nothing’s going to be done. I mean, who’s got $10,000?

They really should have it [bariatric surgery] here because a lot of people who live here can’t afford to fly away to have it done. Yes, [provincial medical plan] will cover the surgery but they don’t cover transportation costs. Once you have the surgery, you have to stay in the area for two weeks. Are they going to pay for your hotel if you don’t have family up there? There’s so much financial burden that a lot of people – this might sound mean – but if you’re not on social services or you’re not rich, what if you’re just in that low-income bracket where you’re still considered the working poor, and you can’t get the extra help from social services or the government.

Others talked about acquaintances that had been able to afford to pay for the procedure. This socioeconomic inequity resulted in high levels of frustration for some participants. One participant said:

It has been really, really frustrating. I cannot stress how frustrated I've been, especially when I have seen friends away being able to get the surgery, and me not be able to get it unless I could afford to go away and pay for it. That part has been really upsetting because they have had success and I am watching them have success away, but I can't access it myself.

For those for whom publicly-funded surgery was the only feasible option, the wait could be very long. The following participant eventually waited ten years from the time bariatric surgery was recommended to her until she was scheduled to undergo the procedure:

They put me on the waiting list. … [The surgeon] told me to just keep calling his office because they were waiting for government funding. So every month I used to call and then it went to every two months, then every three months, and then, I don’t know, maybe two or three years afterwards I just quit calling.

### Perceived regional inequity

Regional inequity was a second concern that surfaced in participants’ accounts. The “official” wait list for bariatric surgery in NL began in May 2011; however, 50% of the participants had been waiting > 5 years to access bariatric surgery in NL or another province. Many participants had considered or explored the possibility of having bariatric surgery done elsewhere in the country [funded by the participant’s province of residence, NL] only to be discouraged by the long wait times in other provinces and the preference of offering the surgery to others residing in the province first, rather than on a first-come first-serve basis. One participant had been waiting for bariatric surgery for 6 or 7 years. She looked into both options but decided to wait until it was payable by her own provincial medical care plan.

I was always looking, I contacted Nova Scotia [another Canadian province], because I knew it was payable there, and I asked if I could get in, but it was a 7-year wait. I contacted Montreal [city in another Canadian province] to see if I could get there and it was a long wait there. I mean MCP would pay for it if I had gone to another province, but the thing is, it’s the wait, because obviously they are going to take their own patients and their own people before they take anybody else, right. So it’s the wait time. I even had a friend that went to Mexico and had it done, and I was even thinking about that but, you know, it costs a lot of money.

Another participant originally planned to undergo the surgery in another province, but found the travel required to be prohibitive. *“I was initially approved through MCP to go to New Brunswick [another Canadian province] to have [Surgeon] do the surgery. So I went to see him twice, but it’s just hard flying back and forth all the time”.*

Participants also pointed to regional inequity within the province, particularly the time and cost of travel from distant parts of the province to the one urban centre where bariatric surgery is offered. Accessibility of the multidisciplinary bariatric clinic added to the financial burden of several participants. One participant commented.

It is a long drive, and it’s expensive. You got to pay for your gas and you got to have at least one night at a hotel. If I’m driving by myself I need two because I can’t do it back and forth in two days. So it’ll cost probably close to $1,000 by the time you do hotel, meals, gas, and whatever, but what option is there? Even to go see [Surgeon] for the first time it cost me, I’m sure, $700 or $800. The flight alone to go in at that time of year was $600 and something. But what do you do? What do you do? MCP covers stuff, but it doesn’t cover anything like that. … You pay it yourself. Then, of course, you feel guilty because if you’re going to spend $1,000, you should put it towards your kids.

Another participant stated.

The only problem I’m having with the whole ordeal is the travelling back and forth… So for a two-day trip, I’m looking at approximately $600. So it’s a day down there, go to my appointment, and then a day to come back. Then I also have to find someone to come with me so that’s extra expenses for me as well. So I got another three trips before I get my surgery. So that’s another $1,800. I spent $1,200 already… That’s going to be five trips to [city] with no surgery, and a final trip to [city] for the surgery. So that’s a lot of money. It’s going to be about $4,000 by the time I actually get the surgery.

### Perceived waitlist prioritization inequity

Although excited about their acceptance as candidates for surgery, the waiting period was described as stressful, anxiety provoking, and frustrating. One candidate put it this way *“I’m cautiously optimistic. I want to believe that it’s going to happen and that it’s going to happen sooner rather than later. But based on history, I’ve been disappointed. So I want to be really positive and optimistic, but I’m cautious. I don’t want to get my hopes up to high. No, that’s a lie. My hopes are already high. I don’t know who I’m fooling”.* Another participant who had been waiting a year stated *“I was a little excited that I found out that I was a candidate for it [surgery]…the excitement kind of wore off. I’m still glad that I’m going to be getting the procedure and whatnot, but it’s kind of hard to hold your excitement for years and months”.*

Participants’ accounts pointed to issues of inequity in the prioritization of the bariatric surgery waitlist. Anger and frustration were expressed towards the health care system for the lack of priority for bariatric surgery and subsequent long waiting times. Participants were exasperated with the lack of information concerning their position on the waitlist and the timeline for surgery. Many felt uncertainty, as if their lives were placed “on hold”, making future plans an unlikely possibility.

One participant, an unemployed labourer in his 20s commented.

What I am supposed to do now? My life is put on hold for this surgery! I want to get this surgery. I want to lose the weight. I want to go back to school. I want to go away to work. I want to get the money so I can come back and finish my house and so on so I can go on and live and be happy… It just seems like a long time to wait…Now it’s just a waiting game.

Although some participants recognized the need to work toward healthy living during the waiting period and were highly motivated to lose weight in preparation for surgery, the uncertainty of when surgery would be scheduled made it difficult to stay focused and sustain momentum. One participant shared.

I could be waiting another year. I don’t know. Not knowing how long you’ve got to wait…when you’re trying to work towards a certain goal and you don’t even know when it’s going to be, it’s hard to stay focused. It’s hard to get motivated and stay focused.

Others felt that their health had been negatively impacted during the long wait:

It is very frustrating … If I was treated eight years ago, I probably wouldn’t have as many issues as I do now. … I need the surgery as a life-saving surgery to me. … I wish they would recognize that fact and give it at least some priority, to get these surgeries done.

Although participants understood that there were others waiting in line for surgery and were willing to take their turn they voiced confusion and a lack of understanding of the waitlist process. Many participants were upset with how patients were being prioritized to receive surgery. While some participants suggested that patients with higher BMIs with more burden of disease should be given priority access to weight loss surgery, others believed it was important to perform surgery on patients’ with lower BMIs. A couple of participants voiced their frustrations and commented thus *“I figured people with higher BMIs would go first because of the fact they might not make it during their wait for surgery”.* One participant, a disabled homemaker in her 50s voiced the following.

I know people who had the surgery that didn’t have really too many problems … Then when you see that and think, well, I’m here suffering and these people are getting the surgery done, and it’s really for people who are in need of it…. It’s hard to sit back and watch it.

A disabled senior narrated.

When is the day going to come? Then you find out that people are being done that weren’t even on the list, weren’t even thought of before I was…I’m 100 pounds overweight…compared to the others [pause] and looking at my medical history. I’m [states age], how long can I wait?… I want to get it done before it is too late…I hope that it is not too late.

Another participant presented a contrasting perspective.

The way that they’re doing them now is purely based on BMI. I don’t think that is appropriate at all… They told me it was completely – the priority list -was completely based on your BMI. For me, personally, I don’t have any of these other factors that most people have: I don’t have diabetes, I don’t have high blood pressure, I don’t have sleep apnea, nothing that most of these other people have. To me, I think, that combined with my age and my lifestyle and everything like that combined should put me in a different place on the waiting list versus somebody who might have a BMI of thirty-six or thirty-seven… who has no interest in going to the gym, who has diabetes.

Participants waiting for surgery communicated how they handled the wait time. One participant stated,

Other than the nervous part of it, the actual surgery, I’m very excited about what’s going to happen because it’s been a long time waiting for it, waiting for change…I did have a slight moment or day, I should say, where I was like: Am I making the right decision: It was like crap, I’m having surgery and it’s major surgery; Am I doing the right thing? I weighed it out. Okay, you’re overweight. You can’t do what you want to do. You may die prematurely because of it. Or you suck it up, you do what you have to do, get healthy, and live a happy life.

Another individual who had an anticipated 12 to 15 month wait for surgery started the required liquid diet to lose weight, but expressed concern about what would happen if he reverted back to his old eating habits because of the long wait. He shared.

So I’m taking all the steps and I’m taking what they say very seriously. I’m doing what I can as early as I can to make myself more prepared for when it actually comes time to get everything done…I know I don’t have to get myself ready right now but I want to. So I just got to keep in the mind frame that I want to be ready for the surgery. The way I see, they could call me tomorrow and say “Come on down”.

Another revealed

I’ve been dealing with it a lot better. Like I said, at first it was tough. Lately it’s kind of like I’m less stressing so much about the weight, and now I’m more thinking about things I’m going to have to do after and ways to do it right. I’m trying to prepare myself for that. The only thing I’m struggling with now is trying not to gain anything because I’m petrified that when I go see [Doctor] he’s going to say “You got five pounds on since the last time you seen [Nurse Practitioner] and we’re not doing you. Now that might never happen, but in my mind that’s there. That is what’s bringing me down lately.

One male participant narrated, *“It’s a waiting game. I’ve been waiting for a long time…I’m pretty positive. I’m not getting depressed or anything”* but also expressed thoughts about what could happen to him: *“My cholesterol could go crazy, my blood pressure could go crazy, diabetes could set in, or any of that kind of stuff. I’m not worried about that stuff now, but it will be an issue. Heart attack or whatever”.*

### Participants’ perceptions of supportive measures to improve the waiting experience

When asked what the health care system could do improve care for patients while waiting for bariatric surgery, participants described what they perceived would be helpful and practical support during the waiting experience. One participant suggested that patients should be given more information about the waiting time for surgery, because waiting without information was the most frustrating part of the experience*. “Give us some hope, because that’s the most frustrating, when you can’t get any information. That’s the most frustrating part of it, waiting and not knowing anything…it would be 100% less stressful if you could just know something”.* Participants suggested that the health care system should provide a regular contact person within the multidisciplinary bariatric surgery clinic who could give them information about the expected waiting time, their place on the wait list and explanations for delays. Fears were expressed that the surgery would never happen, they would not live long enough to have the surgery, or they would fall through the cracks and get lost in the system. Regular contact was wanted in order to feel they were not lost in the system.

I don’t know when it’s going to happen, if it’s going to happen. Maybe I have fallen through the cracks, or maybe they have forgotten me… I do phone every once in a while to the receptionist just to see if there’s any update and there isn’t because they are still so backlogged…I do think that maybe if there was some way they could send you an update every once in a while to say that they haven’t forgotten you, or that sort of thing, because I feel that it is easy for people to forget me. I don’t feel that I matter.

The need for counseling for individuals and their families while waiting for surgery was expressed by several participants. Participants revealed the need for psychological counseling. While some participants recommended that it would be a good idea to bring the spouse/partner into the process early to learn about the surgery along with the patient others participants recognized the need to involve the entire family in the counseling process. The following participants’ comments summarize some of the perceptions about the need for counseling.

I think maybe some psychological counseling and maybe some information on exactly what you’re going through from the time you have to start on a liquid diet and then what you will be going through when you have your surgery and what’s going to happen afterwards. That would be very helpful, I think, to a lot of people. I know it would be to me.

How do I explain it [surgery] to my children? Maybe if there was a family group thing…the type of thing you could bring your husband …then there would be a special time in the session for the children so that we could better explain it to the kids. Either way, your family is involved anyway. If you’re having this surgery it’s something you’ve obviously discussed with them because it’s a big, life-changing possibly life threatening surgery…It’s not just counseling for the patient, but for the family, too.

One participant spoke about feeling mentally exhausted thinking about her upcoming surgery. She narrated *“I actually said to [person] one day, I think I’m going to need counselling. She said “Why”? I said because I can’t think straight half the time because this is on my brain 24/7, this whole surgery thing”.*

Participants also spoke of the economic burden of travelling back and forth to the multidisciplinary clinic for regular appointments. For example, one man stated *“I’m on a very, very restricted budget right now. I get less than $1,000 a month”* and offered practical suggestions that included the scheduling of pre-surgery assessments in the other health regions that would help minimize economic hardship associated with travel, lodging, food, and loss of income.

Other recommendations provided by participants included the establishment of a support group, access to a dietitian to help the prevent weight gain during the waiting period prior to surgery, using patient mentors during the waiting period to help support patients, and providing more resources (e.g. bariatric specific educational tools and more information about what to expect after surgery), among others.

## Discussion

In the current study participants gave historical accounts of their weight histories and spoke candidly about choosing bariatric surgery as a treatment option for obesity and the decision-making process involved. After careful deliberation of the pros and cons of undergoing bariatric surgery as a “last resort” to stop the vicious cycle of weight loss followed by weight regain due to a perceived loss of control over eating, participants were surprised about the length of time it would take to have this “life-saving” surgery.

Perceived inequity was considered a barrier or challenge for participants seeking this treatment option and for achieving their goal of returning to a normal life, living a “transformed life” or starting a “whole new way of living”. Participants spoke directly to three important areas of perceived inequity related to waiting for bariatric surgery: socioeconomic inequity, regional inequity, and inequity related to waitlist prioritization. Health system level factors including the lack of availability of bariatric surgical services and individual level factors related to the financial burden associated with accessibility of the existing service were viewed as impediments or barriers.

Participants’ accounts of the physical and psychosocial consequences of waiting for surgery provide an understanding of the impact of waiting for this surgical procedure. The longer the waiting period the more difficult it was for participants to stay motivated and engaged in maintaining their current health as they prepared for surgery. Participants recognized the need for and advocated the prioritization of patients waiting for bariatric surgery as a means of addressing the growing demand for this service on the health system.

Only a few studies have focused on the experience of waiting for bariatric surgery from the patients’ perspective [31-36]. Several researchers have determined that psychological barriers add to the burden of waiting for surgery and pose as deterrents to maintaining or improving health during the waiting period [32,33,35]. Perceived inequity to accessing bariatric surgery was one of the emergent themes in the current study which provides insight into the psychosocial impact of waiting for bariatric surgery when an individual perceives the barriers to access as being outside of their control. The findings highlight the increased emotional and psychological burden experienced by this group of participants. The current qualitative findings add to the existing body of literature. The findings also appear to be aligned with quantitative literature published on the topic of waiting for bariatric surgery.

In Canada, the majority of health care is publicly funded, and the primary objective of the Canada Health Act is *“*to facilitate reasonable access to health services without financial or other barriers*"*[45]*.* It has been estimated that of the estimated population of over 1.5 million Canadians who would be eligible for bariatric surgery, approximately 0.1% receive publicly-funded bariatric surgery each year [20]. While bariatric surgery-eligible Canadians tend to be less educated than the general population, and more likely to be in the lowest socioeconomic tertile, the tiny fraction who receive bariatric surgery are in fact disproportionately wealthier and more highly-educated [20]. Padwal et al. state that there are no immediately apparent reasons for the socioeconomic disparities in access to bariatric surgery and urge further examination [20]. However, socioeconomic inequity in bariatric surgery is an example of a social gradient in health, even in countries with publicly-funded health programs [46]. To borrow from The Marmot Review, a large-scale review of health inequity in England, health is a matter of social justice: health inequities “reflect, and are caused by, social and economic inequalities in society” [46], p. 10.

Regional inequity exacerbates existing socioeconomic inequity in access to bariatric surgery. Wealthier Canadians are better able to access privately-funded bariatric surgery, and in addition, are also better able to afford the cost of travel to other provinces for publicly-funded surgery. Health care in the Canadian federal system is provided by the provinces and territories. Regional and provincial variations in capacity have given rise to extremely long wait times for bariatric surgery (estimated 5 year average) [30], the longest of any surgically treated condition [47]. The estimated demand exceeds potential capacity by over 600-fold [48]. To address the increased demand, several provincial governments (i.e., Alberta, Newfoundland and Labrador, Quebec and Ontario) have increased capacity; however, it will be extremely difficult to meet the growing demand even with a 10-fold increase in the availability of bariatric surgery within the next 5 years [49]. Further, six of the thirteen provinces and territories have no bariatric surgery program within their own jurisdiction [20], so patients from these regions must travel to other provinces for bariatric surgery, however many of these provinces do not accept non-residents because of the length of their wait lists. Within-region inequity is also a concern in a jurisdiction such as NL, where the population is sparsely scattered across great distances and bariatric health services are centralized in one urban location.

Inequity in terms of waitlist prioritization for bariatric surgery is a third concern identified by bariatric patients. This study revealed that a majority of patients described the waiting period as stressful, anxiety provoking, and frustrating. Anger and frustration was expressed towards the health care system for the perceived protracted wait times. Although no published studies were identified that specifically focused on perceived inequity while waiting for bariatric surgery, research has focused on the psychosocial impact of waiting for bariatric surgery [31-33,35] as well as other elective surgeries [29]. As noted above, bariatric surgery has the longest waiting times of any elective surgery [30] and only a tiny fraction of the surgery-eligible population is able to access bariatric surgery [20]. Concerns have been raised regarding potential inequities in access to bariatric surgery [49]. In addition to socioeconomic advantage, bariatric surgery patients tend to be privileged in terms of health status: younger and healthier, in terms of lower risk of comorbidities, than the surgery-eligible population [20,50]. Men appeared to be underrepresented amongst those who have had bariatric surgery [50] or are deemed eligible for surgery [51] Such inequities highlight the need for prioritization of patients on waitlists [47,52]. Research focused on the development and validation of a prioritization scoring system has begun [13].

Finally, this study provided valuable insight into how participants handled the often times protracted periods of waiting. The psychological impact narrated included: coping with food temptations, dealing with the fear of gaining weight, concern regarding the possible development of obesity related comorbidities such as diabetes, hypertension, and or cardiovascular disease, and the fear of prematurely dying or dying while waiting for surgery. The participants helped to identify a number of supports that could be put in place from the patients’ perspectives to improve the waiting experience. During the waiting period most participants sought support from both formal and informal sources. However, the availability of such supports varied markedly and differed by gender [51]. Waitlists for elective surgery will continue to be a reality in publicly funded health systems where demand exceeds capacity. Importantly, the experiences of being on a waitlist, especially for a protracted period of time, may have serious implications for individual health outcomes. Individuals currently on waitlists for bariatric surgery are in an ideal position to help inform decision-makers and policy makers on strategies that may optimize the waiting experience.

## Limitations

This study was based on a non-random sample localized to one bariatric surgery clinic using qualitative methodology. Results are limited by those agreeing to participate. In total, 27 of 51 (53%) eligible patients approached participated in the study. Bariatric surgery candidates who chose not to participate in the study may have different perceptions than those who participated.

## Conclusions

This study provides detailed qualitative analysis of patients’ experiences on a waitlist for bariatric surgery and highlights three areas of perceived inequity identified in patients’ accounts: socioeconomic inequity, regional inequity, and waitlist prioritization inequity. This study also brings attention to the need for a concerted effort to address the growing dissatisfaction of patients accessing bariatric surgery and the perceived unacceptable wait times that arise once the patient is deemed eligible to undergo the surgery. Waiting for surgery is inherent in publicly funded health care systems; however, ensuring equitable access to treatment should be a health system priority. Supports and resources are required to ensure the waiting experience is as positive as possible.

## Competing interests

The authors declare no competing interests with respect to the research, authorship, and/or publication of this article.

## Authors’ contributions

DG and LT developed the study concept. JTN conducted the interviews. JTN and DG performed data analysis/interpretation. DG wrote the initial draft. All authors performed data interpretation and critically revised the manuscript for important intellectual content. All authors approved the final version of the manuscript.

## Authors’ note

Portions of this article were presented at the 2012 Obesity Society 30^th^ Annual Scientific Meeting in San Antonio, Texas.
